# Development of a Genomic Resource and Quantitative Trait Loci Mapping of Male Calling Traits in the Lesser Wax Moth, *Achroia grisella*

**DOI:** 10.1371/journal.pone.0147014

**Published:** 2016-01-25

**Authors:** Jennifer M. Gleason, Yihong Zhou, Jennifer L. Hackett, Bethany R. Harris, Michael D. Greenfield

**Affiliations:** 1 Department of Ecology and Evolutionary Biology, University of Kansas, Lawrence, Kansas, United States of America; 2 Department of Molecular Biosciences, University of Kansas, Lawrence, Kansas, United States of America; 3 Institut de recherche sur la biologie de l'insecte (IRBI), CNRS UMR 7261,Université François Rabelais de Tours, Tours, France; University of Arkansas, UNITED STATES

## Abstract

In the study of sexual selection among insects, the Lesser Waxmoth, *Achroia grisella* (Lepidoptera: Pyralidae), has been one of the more intensively studied species over the past 20 years. Studies have focused on how the male calling song functions in pair formation and on the quantitative genetics of male song characters and female preference for the song. Recent QTL studies have attempted to elucidate the genetic architecture of male song and female preference traits using AFLP markers. We continued these QTL studies using SNP markers derived from an EST library that allowed us to measure both DNA sequence variation and map loci with respect to the lepidopteran genome. We report that the level of sequence variation within *A*. *grisella* is typical among other Lepidoptera that have been examined, and that comparison with the *Bombyx mori* genome shows that macrosynteny is conserved. Our QTL map shows that a QTL for a male song trait, pulse-pair rate, is situated on the Z chromosome, a prediction for sexually selected traits in Lepidoptera. Our findings will be useful for future studies of genetic architecture of this model species and may help identify the genetics associated with the evolution of its novel acoustic communication.

## Introduction

Lepidoptera is the second largest order of insects (after Coleoptera) and is very diverse in ecology, life history and behavior (reviewed in [1]). Moreover, the order includes a wide range of agricultural pests. Numerous fundamental and applied studies of Lepidoptera have addressed questions on development, nutritional physiology, host-plant interactions, migration, mimicry and aspects of anti-predator defense, and mating behavior, especially that which involves female sex pheromones [2]. On the other hand, genetic analyses of these various traits have lagged behind efforts in other insect orders, and in most cases they have only been conducted at the level of quantitative and evolutionary genetics. But several genome projects in recent years (e.g. [3–6]) have opened the possibility of probing the genetic basis of various facets of lepidopteran biology with greater precision.

Here, we present a genomic analysis of sexual and developmental traits in *Achroia grisella* (lesser waxmoth), a pyralid moth species exhibiting highly unusual acoustic communication during pair formation. Whereas males in various species of moths generate sound signals during close-range courtship that follows their arrival adjacent to a pheromone-emitting female [7, 8], *Achroia grisella* is unusual among moths in that males produce a long-range calling song that attracts females up to 1 m distant. Pair formation may proceed by means of the calling song alone [9–11]. Thus, our study was motivated by the twin objectives of 1) adding to the genomic resources available for research on Lepidoptera and 2) identifying the genomic regions involved in the evolution of the novel acoustic and mating behavior in *A*. *grisella*.

*Achroia grisella* are symbionts of the Western Honeybee, *Apis mellifera*, and are now found in most regions of the world where apiculture is practiced [12]. The moth larvae develop on honeybee brood, stored honey and pollen, wax, and organic detritus of bee colonies, particularly those that have already been weakened by other pests and pathogens [13]. *Achroia grisella* complete development in or near the natal honeybee colony, and mating and oviposition normally take place at that site unless its resources have been exhausted. In that case it is inferred that the adult moths disperse in search of another honeybee colony to infest [14].

Laboratory studies conducted over the past 25 years have shown that female *A*. *grisella* prefer certain acoustic features of male song [15–17] and that several of these song features, pulse-pair rate and amplitude, are generally heritable [14, 18, 19]. Moreover, females vary in how they evaluate male song, and aspects of their evaluation protocol are also heritable [20–22]. Several song features evaluated by females are correlated with developmental parameters; these too exhibit significant heritability [18, 19]. Importantly, male song, female response, and developmental parameters are also strongly influenced by environmental variables, e.g. temperature and food availability, and significant genotype x environment interactions occur [14, 21, 23–27]. Recent quantitative trait loci (QTL) studies relying on anonymous AFLP markers have identified genomic regions with major effects on features of male song, female response to and preference for male song, anti-bat defensive responses, and developmental parameters [28, 29].

Our present study has the advantage of using identifiable coding sequences to allow us to infer homologous chromosomes in other Lepidoptera. We sequenced a small EST library to produce SNP markers for *A*. *grisella*. We then used these SNP markers to analyze a large backcross population between two inbred *A*. *grisella* strains, which yielded a set of QTL for sexual and developmental traits. Comparison of our findings on *A*. *grisella* with the *Bombyx mor*i (silkworm moth) genome, confirmed a high level of synteny within the order (cf. [30]). This comparison also indicated that at least one male signaling trait, pulse-pair rate, is linked to the Z (sex) chromosome.

## Materials and Methods

### Moth lines

Two inbred strains of *Achroia grisella* were used in the study. Insects were collected on private land in Lawrence, Kansas (1997) and in Lake Worth, Florida (2003). *Achroia grisella* are not an endangered or protected species, therefore no specific permissions were required for the collections other than the permission of the landowner, which was given in each case.

To establish inbred lines, field collected populations were transferred to plastic boxes containing a standard synthetic diet [11] and reared in growth chambers at 25˚C under a 12:12 hr L:D photoperiod. From each stock population, 100 inbred lines were started with a single breeding pair followed by single brother-sister mating to produce subsequent generations. Each line was inbred for at least 25 generations prior to this study. We chose two lines, Kansas 89 (hereafter, “Kansas”) and Florida 90 (hereafter “Florida”) to serve as the P1 generation. These lines were chosen to represent the two geographic populations and because the lines differed in several song traits [22, 26].

### Construction of EST Library

The Kansas line was used to construct the EST library. Total RNA was extracted using an RNeasy Mini Kit (Qiagen, Valencia, CA) from the heads of eight moths (four males and four females). A cDNA library was constructed using the Creator^TM^ cDNA Library Construction Kit (Clontech, Mountain View, CA). First strand synthesis of cDNA, long-distance PCR (LD-PCR) for synthesis of full-length dscDNA, SfiI digestion and cDNA size fractionation were conducted according to the user manual. LD-PCR was run for 20 cycles. After ethanol precipitation, SfiI-digested cDNA was ligated to the Sfil-digested, dephosphorylated pDNR-LIB Vector provided with the kit. The recombinant plasmids were transformed into Max Efficiency® DH5α^TM^ competent *Escherichia coli* cells (Invitrogen, Carlsbad, CA). Plasmid DNA was isolated using QIAGEN Plasmid Mini Kit (QIAGEN, Valencia, CA), then sequenced using the M13 primer on an ABI 3130*xl* Genetic Analyzer (Applied Biosystems, Foster City, CA) at the University of Kansas sequencing laboratory. Chromatogram files were exported to Sequence Scanner (V.1.0; Applied Biosystems, Foster City, CA) for base calling and removal of poor quality sequences. Vector sequences, adaptor region and poly(A) tails were trimmed from the sequences.

The resulting sequences were examined for redundancy by forming contigs using Sequencher (Gene Codes Corporation, Ann Arbor, MI). Unique contigs were compared against the National Center for Biotechnology Information (NCBI) nonredundant protein database using BLASTX through Blast2GO [31].

### Resequencing in Kansas and Florida Lines

We designed primers for resequencing the ESTs in the Kansas and Florida lines using Primer3plus (http://www.bioinformatics.nl/cgi-bin/primer3plus/primer3plus.cgi). DNA was extracted from adult abdomens using the DNeasy kit (Qiagen). A high-fidelity Taq DNA Polymerase, LongAmp Taq DNA Polymerase (New England Biolab, Inc, Ipswich, MA) was used in all reactions, according to the manufacturer’s instruction. For the primer sets that successfully amplified targeted DNA sequences from both inbred lines, PCR products were purified with ExoSAP-IT (GE Healthcare Life Sciences, Pittsburgh, PA) and submitted to the University of Kansas sequencing laboratory for forward sequencing analysis. DNA sequences generated from the same loci of the two lines were compared pairwise to identify potential SNPs or INDELs (insertions/deletions). To further confirm the sequence variation existing between these two lines, a second round of sequencing analysis (reverse, or a second round of forward sequencing analysis depending on the length of the targeted DNA fragments) was performed to verify the initial results.

### SNP Genotyping

The designed SNPs were used to develop a genetic map based on the Kansas and Florida lines. Virgin female Kansas moths were crossed to male Florida moths and the resulting F1 males were backcrossed to Kansas female moths. DNA was extracted as above from either a half or full abdomen of the resulting backcross hybrid males. Final DNA concentrations of 40–60 ng/μl in 15 μl were used in genotyping.

SNP assay primers were designed based on the Kansas and Florida sequences. SNP sequences were submitted to the online Illumina Assay Design Tool. The 96 highest scoring sequences were subsequently used in SNP genotyping performed using the Illumina BeadXpress platform at the UC Davis Genome Center.

### Construction of the genetic map and comparison to *Bombyx mori*

Of the original SNP sequences used in genotyping, 75 were successfully scored. Using the genotypes of the backcross individuals, a genetic map was constructed using the Kosambi mapping function in MAPMAKER [32]. In this manner, each marker was assigned to a linkage group. The mapped EST sequences were subsequently compared to the genome of *Bombyx mori* using BLASTx on the Silkworm Genome Database (SilkDB; http://silkworm.genomics.org.cn/). The top homologous sequences were used to identify *Bombyx mori* scaffolds and their associated chromosomes for a comparison across species. *Bombyx mori* was used because it is both a moth and has a well-assembled genome.

### Phenotyping for QTL mapping

Prior to DNA extraction for the genetic map, male moths from the backcross populations were phenotyped for two life history characters and three signaling parameters. Development time (DT) was reported as the number of days from oviposition to adult eclosion, and body mass (Mass, ± 0.005 mg) was measured immediately following adult eclosion. The signaling parameters measured were pulse-pair rate (PR), peak amplitude (PA) and asynchrony interval (AI) length, which combined have a major influence on a male’s attractiveness to females in both parental populations [15]. Males produce their ultrasonic song while remaining stationary on the substrate and wing-fanning, which causes a pair of tymbals on the tegulae (sclerotized plates at the base of each wing) to buckle during each upstroke and downstroke. Fanning of the left and right wing is not simultaneous; thus pulses (tymbal buckles) are normally produced in pairs, and AI is the time interval between the onsets of the two pulses. The rate at which the pulse-pairs are produced is PR, and the peak sound pressure level is PA.

We recorded the song of each male moth during the first day following eclosion. Recordings were done in an acoustically insulated chamber kept at 25 ± 1°C and were always done during the initial 6 h of the photoperiodic night to coincide with the normal activity pattern of these nocturnal insects [16]. Males were placed individually in small screen cages (1.5 cm diam., 2.0 cm height) 30 cm away from neighboring cages and separated with acoustic insulation foam to minimize background influence from their neighbors. Male song was recorded using a condenser microphone (model 7016, ACO Pacific; Belmont, CA; frequency response: ± 2 dB from 10 to 100,000 Hz, ± 6 dB from 10 to 160,000 Hz) pointed toward the cage at a standard distance of 10 cm. The microphone output was amplified (model 4012 preamplifier, model PS9200 amplifier, ACO Pacific), filtered (model 3202 variable filter, Krohn-Hite, Brockton, MA) and digitized (National Instruments DAQCard 6062E, National Instruments, Austin, TX). A 1-s sample of digitized song was saved using Batsound 4.0 software (Petterson Elektronik AB, Uppsala, Sweden). For each male, we calculated the pulse-pair rate (PR), mean peak amplitude (PA) and asynchrony interval (AI) using a custom-written computer script in Spike2 (Cambridge Electronic Design, Cambridge, UK). PA was calculated in arbitrary linear units for each pulse, which were averaged over the 1-s recording sampled.

### Statistical analyses of phenotypes

Moths were crossed and phenotyped in two blocks (two different years). Comparisons were made for each phenotype between years using t-tests in R. For traits that differed significantly between the years, Z-scores were used in subsequent analyses. Pearson’s product-moment correlations and covariances among phenotypes were also calculated in R.

### QTL mapping

The genetic map was imported into QTL Cartographer version 1.17j [33]. Analyses were conducted using composite interval mapping (CIM). For CIM, window sizes of 1, 5, and 10 cM were tested with all significant markers obtained from interval mapping; the results were not significantly different with window size and only the results using a 5 cM window are presented here. Epistasis was tested by using a general linear model with nearest marker loci as covariates.

## Results

### EST Library

The EST library was constructed from head tissue of the Kansas line. A total of 594 clones were sequenced, yielding 352 contigs. Fifty-seven contigs were redundant with 2–94 sequences per contig (mean 5.54 ± 13.08 sd), the remaining 295 were singletons for a mean of the entire set of 1.70 ± 5.40 sequences per contig. Contigs were annotated using Blast2GO [31] using BLASTx resulting in 233 with hits and 119 without hits. Eighteen sequences were subsequently removed from further analysis because 16 were mitochondrial and two were bacterial. Of the remaining 334 contigs, 215 had blast hits. Contigs without hits were significantly shorter (486.48 ± 239.50 bases) than contigs with hits (637.30 ± 258.65 bases; t-test, P<<0.0001).

Percent nucleotide similarities of BLASTx hits ranged from 48.0% to 100% (mean 77.10 ± 13.27%) and observed E-values ranged from 2.4 x10^-4^ to 10^−180^ (mean 3.74 x 10^−6^ ± 2.08 x 10^−5^) after a cutoff value of 10^−3^ (S1 Fig). BLASTx hits were observed for multiple species but the top hit for 91.6% of the contigs with a match was lepidopteran with 4.7% other insect species and 3.7% non-insect species. Of the 3650 total hits, 31.8% were lepidopteran and 56.0% were other insects. The number of hits per contig ranged from 1–20 (the set limit) with a mean of 16.98 ± 6.46.

Gene ontology (GO) annotations were found for 165 of the 215 sequences with BLAST hits. The mean number of GO IDs per contig was 2.76 ± 3.77 IDs (range 0–38). GO predictions included many different biological processes (Table A in S1 File) without particular enrichment in any category.

### Molecular Divergence between Kansas and Florida Lines

To identify SNPs for genetic mapping, we resequenced loci in both the Kansas and Florida lines. We had 2X coverage for each strain for 136 sequences and a total of 32,334 aligned base pairs (bp) ranging from 51 to 1136 bp per sequence (mean ± sd: 461.91 ± 305.22 bp). We identified SNPs and insertions/deletions (indels); 35 sequences (25.74%) lacked SNPs, 89 lacked indels (65.44%) and 32 lacked SNPs and indels (23.53%).

The number of SNPs found ranged from 0 to 30 per sequence with a mean of 3.57 ± 5.01 (Table 1). Transitions significantly exceeded transversions (Table 1; **Χ**^2^ = 8.963, 1 df, *P* = 0.0028) but there was not a difference in the number of A/G and T/C transitions (Table 1; **Χ**^2^ = 2.44, 1 df, *P* = 0.1176). Among the transversions, A/T transversions were overrepresented (Table 1; **Χ**^2^ = 22.34, 3 df, *P<*0.0001); this is reflective of the greater abundance of A and T than G and C in the genome (GC content is about 30%, S.J. Macdonald, pers. comm., which is similar to that found in other lepidopterans, [6]). The overall nucleotide diversity between the Kansas and Florida strains was 0.0087 ± 0.0104, similar to that of other Lepidoptera [34–37].

**Table 1:** Characteristics of SNPs between the Kansas and Florida lines.

	# of SNPs	Mean number SNPs per sequence (n = 136)
All	486	3.57 ±5.01
Transitions	276	2.03 ± 3.00
A/G	151	1.11 ± 1.78
T/C	125	0.92 ± 1.46
Transversions	210	1.54 ± 2.33
G/T	45	0.33 ± 0.70
A/T	82	0.60 ± 1.09
C/G	40	0.29 ± 0.70
C/A	43	0.32 ± 0.67

The percentage divergence based on SNPs is an underestimate given the variation that exists in indels. A total of 87 indels were found with 0 to 5 found per sequence and a mean of 0.64 ± 1.16 per sequence. Indels were variable in length (S2 Fig) ranging from 1 to 272 bp with the majority 1 or 2 bp (47 indels or 54.02%). Arbitrarily treating each indel as an insertion in one line, more insertions exist in the Kansas line than in the Florida line, though the difference was not significant (Table 2; **Χ**^2^ = 0.56, 1 df, *P<*0.4530), nor were the size of those insertions (t-test, *P* = 0.6071).

**Table 2:** Characteristics of indels between the Kansas and Florida lines.

	Total	Kansas	Florida
Number of indels	87	47	40
Maximum insertion length* (bp)	272	272	55
Mean insertion length (bp)	8.30 ± 29.69	9.83 ± 39.12	6.50 ± 10.60
Total insertion length (bp)	722	462	260

*Indels were arbitrarily designated as insertions to enable analysis.

### Genetic Mapping

The SNP analysis allowed for the development of 96 SNP markers that were scored for 576 DNA samples. Of these, 11 markers were discarded because 1) the parental individuals had the same alleles, 2) one of the parental lines produced a null allele, or 3) all individuals were scored as hybrids. Of the remaining 84 markers, two sets of two markers were later identified as being for the same locus, and six sets of two or three loci had exactly the same genotype for all individuals. One in each set was deleted and the final analysis is based on 75 markers.

The mapping population consisted of backcross hybrid males. In two separate years, female Kansas moths were crossed to male Florida moths. Because female moths do not have recombination [38], F1 males were backcrossed to Kansas female moths. Individuals were phenotyped (see below) and subsequently genotyped. Genotyped males included 562 experimental backcross hybrid males and control males: two Kansas line individuals, two Florida line individuals and two hybrid males. All control males were scored in duplicate or triplicate to check for consistency of marker scoring within and between plates. Control individuals were removed from subsequent analysis. Experimental individuals removed from analysis included a duplication, seven backcross hybrid males that failed to be scored for any marker and an individual that consistently scored as homozygous for the Florida alleles, which was not possible for the experimental design. The final data set consisted of 552 backcross hybrid males.

For each marker, the number of individuals scored ranged from 548–552 with a mean of 551.27 ± 1.02 (sd). For each individual, the number of markers scored ranged from 70–75 with a mean of 74.9 ± 0.50 (sd). The markers formed 28 linkage groups with a range of 1–7 markers per linkage group (mean 2.68 ± 1.17 (sd) markers/linkage group). The number of linkage groups indicated that markers were not found for all chromosomes because the estimated haploid chromosome number for *Achroia grisella* is 30 [28]. The mean distance among markers in linkage groups was 4.72 ± 10.18 (sd) cM, thus recombination among markers was low and variable, but similar to that found for this species in another QTL study using AFLP markers [28].

Markers were examined for Mendelian segregation. Ten markers had an excess of heterozygotes by a Chi-Square test (*P*<0.05), though none was significant after Bonferroni correction (for 75 tests, this corresponds to *P*<0.00067); these markers formed two linkage groups with seven in linkage group 2 and three in linkage group 9. Because segregation distortion was found for linkage groups and not for individual markers, this implies that biotic factors were responsible and not that the markers were scored incorrectly. Segregation distortion, particularly for excess heterozygotes may be favored in mapping populations when homozygous alleles decrease fertility and viability [39–41].

For the markers in linkage group 9, all had a significant excess of heterozygotes in the year 1 data but not year 2 data (Chi-square test, P<0.05) though none was significant after Bonferroni correction. The markers in linkage group 2 had a significant excess of heterozygotes in both years (Chi-square test, P<0.05) but again, none was significant after Bonferroni correction. Comparing between the years, two markers in linkage group 2 were significantly different in scoring for heterozygotes and homozygotes (two-tailed Fisher’s Exact test, P = 0.05) and both in linkage group 19 homozygotes (two-tailed Fisher’s Exact test, P<0.01), though again, these were not significant after Bonferroni correction.

The nucleotide sequences of the 75 markers were compared to the *Bombyx mori* genome using BLASTx to identify predicted proteins and then infer syntenic chromosomes from the scaffolds of those proteins. Of these, 46 markers matched *B*. *mori* sequences. In every case in which more than one marker per linkage group was homologous with a *B*. *mori* sequence, all of the matches were on the same chromosome in *B*. *mori* (Table B in S1 File). In this manner, the syntenic chromosomes for 20 linkage groups were determined (Table B in S1 File). In one case, a linkage group with only one marker (#24) matched the same *B*. *mori* chromosome as another linkage group with two markers (#18). The scaffolds to which these two matched were on the tip of *B*. *mori* chromosome 24 (linkage group #24) and many scaffolds away (linkage group #18). No other linkage groups mapped to the same *B*. *mori* chromosomes. Of note, our linkage group 1 is homologous to the *B*. *mori* Z chromosome.

### QTL mapping

Phenotyping was conducted in two different years. The Kansas and Florida lines differed in DT, Mass and PR (t-test, P<<0.001 for each) but not PA or AI (t-test, *P*>0.05, data not shown). Hybrid individuals were variable in being similar to each line but were different from both in Mass, PR and PA (Table 3). For these traits, inbreeding depression in the parental lines may be playing a role because Mass is higher in hybrids (F1 generation) than parents in both years, PR is faster in year 2 and PA is louder in both years. This pattern of inbreeding depression and recovery in hybrids has been seen in other studies of *A*. *grisella* [27].

**Table 3:** Phenotypic values for all traits in parents, F1 hybrids and backcross individuals.

		DT (days)		Mass (mg)		PR (msec)		PA		AI (μsec)	
Year	Males	N	mean ± s.d.	N	mean ± s.d.	N	mean ± s.d.	N	mean ± s.d.	N	mean ± s.d.
1	Kansas	96	42.85 ± 2.61	95	12.26 ± 1.50	95	75.83 ± 4.89	95	69.99 ± 12.79	95	690.01 ± 396.88
	Florida	97	43.52 ± 1.86	97	16.95 ± 1.60	97	71.36 ± 4.53	97	63.64 ± 12.35	94	639.68 ± 407.12
	Hybrid	71	41.21 ± 2.31	70	17.31 ± 1.24	66	74.50 ± 6.00	67	86.90 ± 15.71	57	747.61 ± 347.50
	Backcross	283	42.12 ± 3.47	283	14.35 ± 1.97	283	75.18 ± 5.99	283	75.17 ± 16.04	283	745.17 ± 366.51
2	Kansas	291	56.26 ± 8.28	288	11.74 ± 1.74	277	75.30 ± 5.68	277	68.18 ± 12.50	277	665.89 ± 367.36
	Florida	181	42.57 ± 6.43	180	15.31 ± 2.65	161	74.30 ± 6.43	95	69.99 ± 12.79	161	650.68 ± 427.73
	Hybrid	54	46.22 ± 5.64	54	15.74 ± 2.23	52	71.85 ± 4.45	52	73.48 ± 13.29	52	767.56 ± 355.17
	Backcross	269	44.53 ± 5.23	269	11.88 ± 1.76	269	75.18 ± 5.33	268	64.99 ± 13.03	269	727.17 ± 398.56

**Fig 1:**
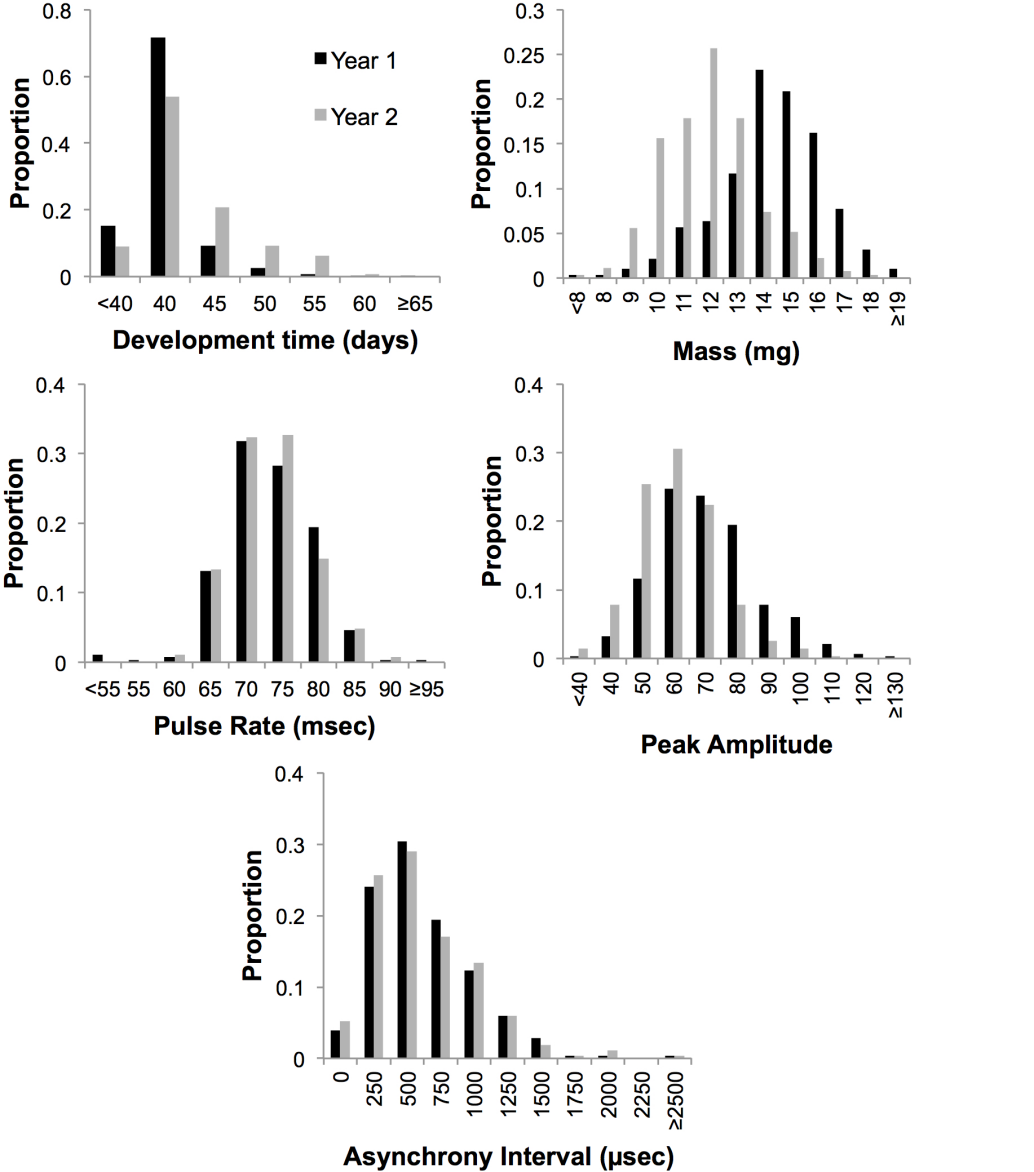
Distributions for the five traits among the mapping populations, separated by year of measurement. The sample sizes, traits means and standard deviations are given in Table 3.

Backcross individuals were phenotyped and genotyped in two different years. Significant differences in backcross populations existed between the years in DT, Mass and PA (Fig 1, t-test, P<<0.0001 for all). To handle these differences, Z-scores (standard scores) were calculated and used for those traits in QTL analysis. DT was negatively correlated with Mass (Table 4). Mass was positively correlated with PA indicating that large moths sang more loudly than small moths (see [18]). No other traits were correlated after Bonferroni correction for multiple comparisons (*P* = 0.005).

**Table 4:** Correlations and covariances among phenotypic traits.

	Development Time	Mass	Pulse-pair rate	Pulse Amplitude	Asynchrony Interval
Development time		-0.1753*	-0.1318*	-0.1058*	0.0982*
Mass	-0.1755		-0.1033*	**0.4071**	0.0745
Pulse-pair rate	-0.2776	-0.5873		-0.0667	-0.1079*
Pulse Amplitude	-0.1061	0.4082	-0.3809		0.0455
Asynchrony Interval	36.6383	28.7866	-234.6651	17.4421	

Correlation coefficients are above the diagonal and covariances below. Correlation coefficients in bold are significant with Bonferroni correction (*P*<0.005) whereas * indicates significance at *P*<0.05.

Results from composite interval mapping (CIM) varied little with window size so all results presented are for a window size of 5 (Table 5, S3 Fig). At the *P* = 0.05 significance level, 1000 permutations of the data set indicated threshold likelihood ratios of 10.63 (PR) to 11.98 (DT); most QTL listed in Table 5 exceed this threshold, the exception is the second PA QTL, which is not significant with a window size of 5, but is with a window size of 10. The other QTL that varied with window size was the second for DT, which was not significant with a window size of 1. No QTL were found for AI. Because not all markers were associated with linkage groups, CIM was not possible for those individual markers. In those cases single marker regression was used and none had a significant association with any of the traits.

**Table 5:** Quantitative Trait Loci mapping results^a^.

Trait	QTL number	Linkage group	Homologous *Bombyx mori* chromosome	Position (cM)	Likelihood ratio	Additive effect	Percentage phenotypic variance explained
Development Time	1	2	15	4.72	56.68	0.61	9.17
	2	5	8	0.01	12.89	0.28	2.00
Mass	1	2	15	2.67	33.34	-0.47	5.42
	2	7	Unknown	0.01	11.58	-0.27	1.83
Pulse-pair Rate	1	1	W	0.75	14.54	-1.77^b^	2.42
	2	7	Unknown	0.01	11.68	1.58^b^	1.93
Pulse Amplitude	1	2	15	5.08	18.94	-0.37	3.35
	2	9	11	6.55	10.63	-0.28	1.86

^a^Shown are the QTL locations with a window size of 5 cM. With a window size of 1 cM, the second Development Time QTL is not significant. Although not significant here, the second Pulse Amplitude QTL is significant with a window size of 10 cM. No other QTL varied with window size. Likelihood ratio significance thresholds for *P* = 0.05 were 11.98 (Development Time), 10.80 (Mass), 10.63 (Pulse-pair Rate) and 10.66 (Pulse Amplitude). No significant QTL were found for AI, which had a significance threshold of 10.95.

^b^With the exception of Pulse-pair Rate (PR), the other analyses were performed with Z-scores. For PR, the additive effects are directly interpretable with effects occurring in milliseconds.

Individual QTL explain from approximately 2 to 9% of the phenotypic variance, thus not a large proportion of the existing variation in the mapping population. The largest proportion explained was 11% over the two QTL for DT. Additive effects for DT and Mass, which are negatively correlated, were in opposite directions. Similarly, additive effects for DT and PA, also negatively correlated, were in opposite directions (thus Mass and PA are in the same direction). All three of these traits were associated with QTL on linkage group two, though DT and Mass had additional QTL on other linkage groups (5 and 7, respectively). No epistatic interactions were detected for any QTL for any of the traits.

For some of the linkage groups, the homologous *Bombyx mori* chromosome was identified (Table 5, Table B in S1 File). Of note, PR, a sexually selected trait, is associated with the Z sex chromosome. As little is known about the genetic basis of calling in acoustic moths, no particular insights are available for associations with the other chromosomes.

None of the significantly correlated traits shared QTL locations, although Mass and PR were negatively correlated as has been observed before [18]; the correlation was not significant after Bonferroni correction. Mass and PR shared a QTL with effects in opposite directions. The traits with the highest correlation, Mass and PA, did not share any QTL, at least with the limits of detection of this study.

### Synteny

In all, 20 linkage groups were matched to a *B*. *mori* chromosome (Table B in S1 File). For two sets of two linkage groups, the same *B*. *mori* chromosome was identified (linkage groups 9 and 15 correspond to *B*. *mori* chromosome 11; linkage groups 11 and 25 correspond to *B*. *mori* chromosome 13). In both of these cases, the homologous scaffolds are far apart on the chromosomes. In general this indicates that we did not have markers on all chromosomes.

For seven linkage groups, the homolog for more than one marker was found in *B*. *mori*; in all of these cases the same *B*. *mori* chromosome was identified. In two cases (linkage groups 2 and 17) where markers corresponded to more than two scaffolds, the marker order within a linkage group was the same as the scaffold order within the *B*. *mori* chromosome, indicating that synteny has been maintained across a large phylogenetic distance.

## Discussion

Our EST project provided some insights into the existing genetic variation within *Achroia grisella*, synteny among Lepidoptera, as well as the genetic basis of several developmental and behavioral traits in an acoustic moth that has been extensively studied with respect to sexual selection (e.g. [15, 26, 42–44]). As genome resources are further developed (a genome project is currently underway, S.J. Macdonald, pers. comm.), the lesser waxmoth may become an ideal organism for the study of the genetics of signaling traits.

### Population genetics

*Achroia grisella* has been transported around the world with apiculture and is an economic pest, particularly in stressed colonies of honey bees [45]. Little is known about how the species moves among hives in a local area. Males call for females in the vicinity of the natal hive [9], thus dispersal is expected to be limited to the situation when a hive declines, which may have been more common under natural (non-apicultural) conditions. Development of markers for measuring F_st_ and other population genetic parameters should be easy given the level of nucleotide diversity between the Kansas and Florida lines, which was on the order of that seen in other Lepidopterans [34–37]. This opens up the possibility of studies of the population genetics of the species to parallel evidence for the role of environmental heterogeneity in maintaining variation in song characters in this species [26].

### Synteny

Because it was possible to identify homologs of many of the EST sequences in the *Bombyx mori* genome, the corresponding chromosomes could be identified for many of our linkage groups. *Achroia grisella* has 30 pairs of chromosomes [28] while we had 28 linkage groups, though if synteny holds, these may represent at most 27 chromosomes because two linkage groups map to the same *Bombyx mori* chromosome. In addition, many of our linkage groups are represented by single markers and may not represent independent chromosomes. Nonetheless, if a linkage group had multiple markers, all of the markers with homologous *B*. *mori* sequences were from the same chromosome and in the same order as found in *B*. *mori*. Lepidopterans are known to have high degrees of synteny among chromosomes, even in species that have diverged over 100 MYA [46–48], though most of the synteny may be on the level of the chromosome (macrosynteny) with multiple local rearrangements contributing to loss of gene order synteny (microsynteny), even in more closely related species [49]. Our results, in comparison to *B*. *mori*, which diverged from pyralid moths such as *A*. *grisella* over 100 MYA [50], indicate that macrosynteny has been preserved because mapped homologous genes are located in the same linkage group. As more lepidopteran genomes become available, further comparative genomics will be possible for comparisons of not only synteny but also sexually selected traits.

### Developmental traits

DT and Mass were negatively correlated, indicating that larvae that grew fast had a larger adult mass. Although development time and adult mass are often positively correlated, with long growth periods resulting in large individuals [51, 52], a negative relationship as found here has been observed in other Lepidoptera [53]. In this study, QTL for DT, Mass and PA, co-localized to the same linkage group; because these traits were correlated, it is tempting to infer that the same or linked genes may be at work here. Co-localization of QTL were not found for these traits by Limousin *et al*. [28], though Alem *et al*. [29] found linkage for one QTL each for Mass, DT and PR.

### Signaling traits and sexual selection

All of the QTL in this study explain a small amount of the phenotypic variance, implying that these traits are polygenic. In other studies with smaller sample sizes, though much larger marker set [28, 29], more QTL were found for these traits; one study [28] had a QTL for AI whereas the other [29] did not. Because the other studies used AFLPs for markers, we cannot directly compare the results here with those studies because we do not know which linkage groups are homologous.

Given that synteny is supported, the presence of a QTL for a sexually selected trait (PR) on the Z chromosome deserves further investigation. Like all Lepidoptera, *A*. *grisella* females are the heterogametic sex with ZW sex chromosomes and males have homogametic Z chromosomes. Sex linkage of traits in ZW systems are predicted to be conducive to Fisherian runaway sexual selection [54, 55]. Other studies of the genetics of this trait in this species have not been able to distinguish sex chromosomes from autosomes [28, 29]. Thus our finding is the first indication of sex linkage for this sexually selected trait.

It is intriguing that no QTL were found for AI, a trait that may be more limited by physiological structure than genetic variation. Part of the reason may be that the parental lines did not differ in this trait. However, because transgressive segregation is so prevalent [56], within species studies can identify QTL in backcross populations with wide distribution in a trait as has been accomplished in other studies (e.g. [57]).

AI is produced by an asynchrony in the movement of the two wings: males do not sing symmetrically with both wings moving simultaneously. A natural population of males varied in having no AI to an AI of around 2500 μsec with a mean and standard deviation of 500 ± 407.4 μsec [15]. Females have preferences for the AI that exceed the mean [15]. Our moths sang within the natural range, varying from 0 to 2542 μsec, which is similar to that observed by Limousin *et al*. [28], who found a QTL, but wider than that of Alem *et al*. [29], who did not. AI may be influenced by male size with larger males having a larger AI [18], but if this were the case, then we would expect a higher covariance and potentially coincident QTL. A previous study [19] found low and nonsignificant heritability for AI, which is consistent with an absence of QTL.

As our genetic resources increase for this non-model organism, the ability to resolve the linkage relationships among these traits will increase. In addition, this species is ideal for studying not only the production of a signal (calling song), but also the response to song because females readily respond to playback of calls (e.g. [15, 58]). In the other two QTL studies [28, 29], the female response was also mapped and found to not be linked to male calling traits or bat defense response [29], indicating that if calling song is an example of sexual selection through sensory exploitation of the ability to detect bat calls, the responses are now unlinked. Further exploration of this system and genetics of the traits involved in sexual signaling and preferences should provide insights into the evolution of female preferences.

The genetic architecture of calling song parameters is clearly polygenic, as has been found for other insects with acoustic signaling, particular orthopterans and *Drosophila* (e.g. [59, 60, 61]). This contrasts with other modes of signaling, particularly pheromonal signaling, which can be influenced by few, or even single, large effect loci (e.g. [62, 63]). As more genomes in Lepidoptera become available, comparisons with other species that have tympanal ears, but lack acoustic signaling, or have independently evolved acoustic signals (e.g. [64]) may be particularly informative to understand the evolution of novel traits.

## Supporting Information

S1 Fig Distribution of E-values from BLASTx hits. The upper cutoff was 10^−3^, thus the first bin is not as large as the others. The last bin includes everything lower than 10^−170^.
(TIFF)

S2 Fig The distribution of indel lengths between the Florida and Kansas lines. Indels were arbitrarily designated as insertions if they appeared in a line. A total of 87 indels were found.
(TIFF)

S3 Fig Genetic map and locations of QTL. The linkage groups and the associated QTL for four of the traits are shown. No QTLs were found for asynchrony interval. Markers (ticks) correspond to those listed in Table B in S1 File. Each QTL was associated with an entire linkage group. The number above each linkage group indicates our linkage group designation followed by the corresponding *Bombyx mori* chromosome (U indicates that the chromosome is unknown) determined by homology of markers (see Table B in S1 File for further descriptions). Linkage groups with single markers are not shown and none was significantly associated with a QTL.
(TIFF)

S1 File Table A. GO Slim Processes for the EST library. Table B: Correspondence of *Achroia grisella* markers to *Bombyx mori* scaffolds. (DOCX)
