# Supplementary figures and images for: Development of a Genomic Resource and Quantitative Trait Loci Mapping of Male Calling Traits in the Lesser Wax Moth, Achroia grisella
Source: PLoS One. 2016 Jan 25;11(1):e0147014. doi: 10.1371/journal.pone.0147014 (PMC4726463; doi:10.1371/journal.pone.0147014)

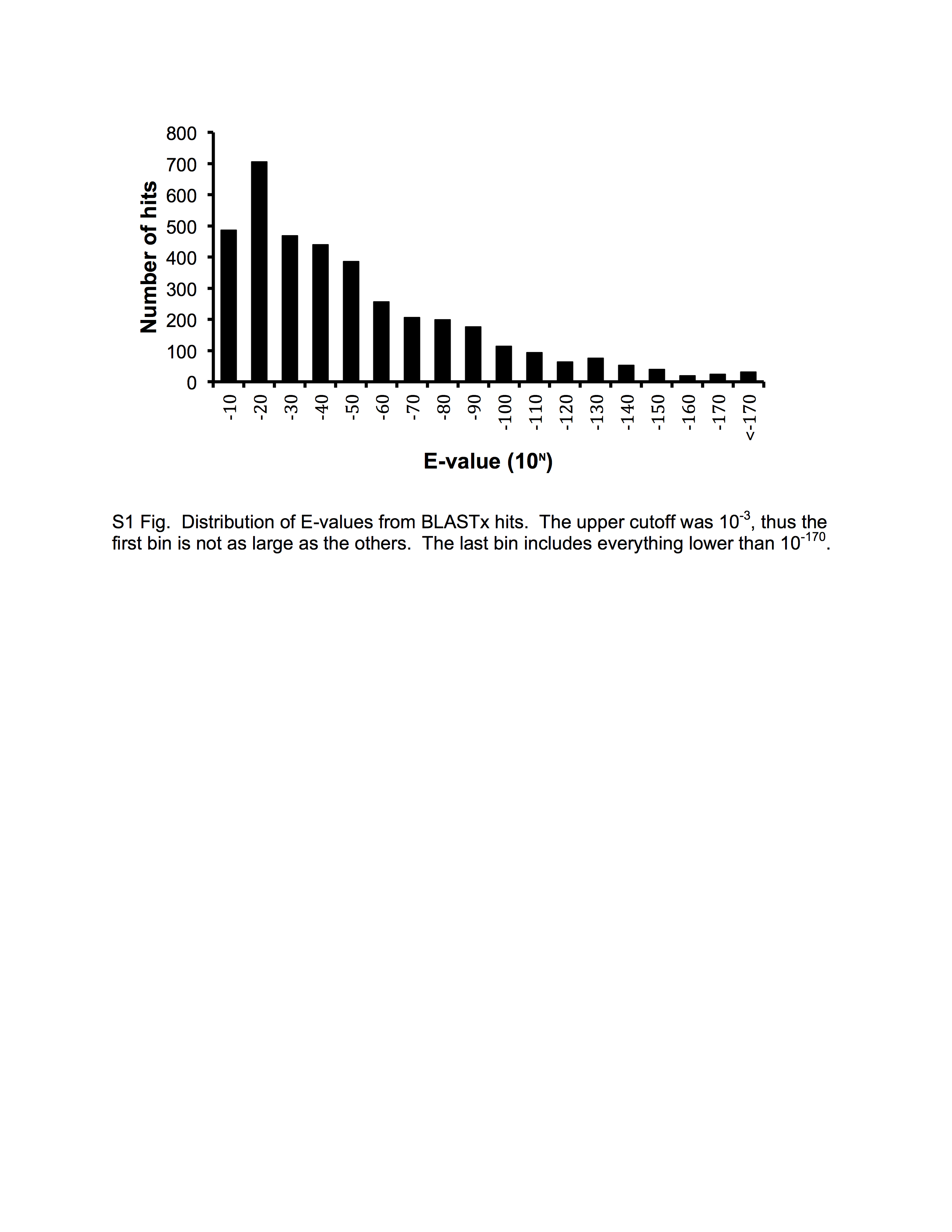

Supplement: S1 Fig — The upper cutoff was 10−3, thus the first bin is not as large as the others. The last bin includes everything lower than 10−170. (TIFF) [file pone.0147014.s001.tiff]

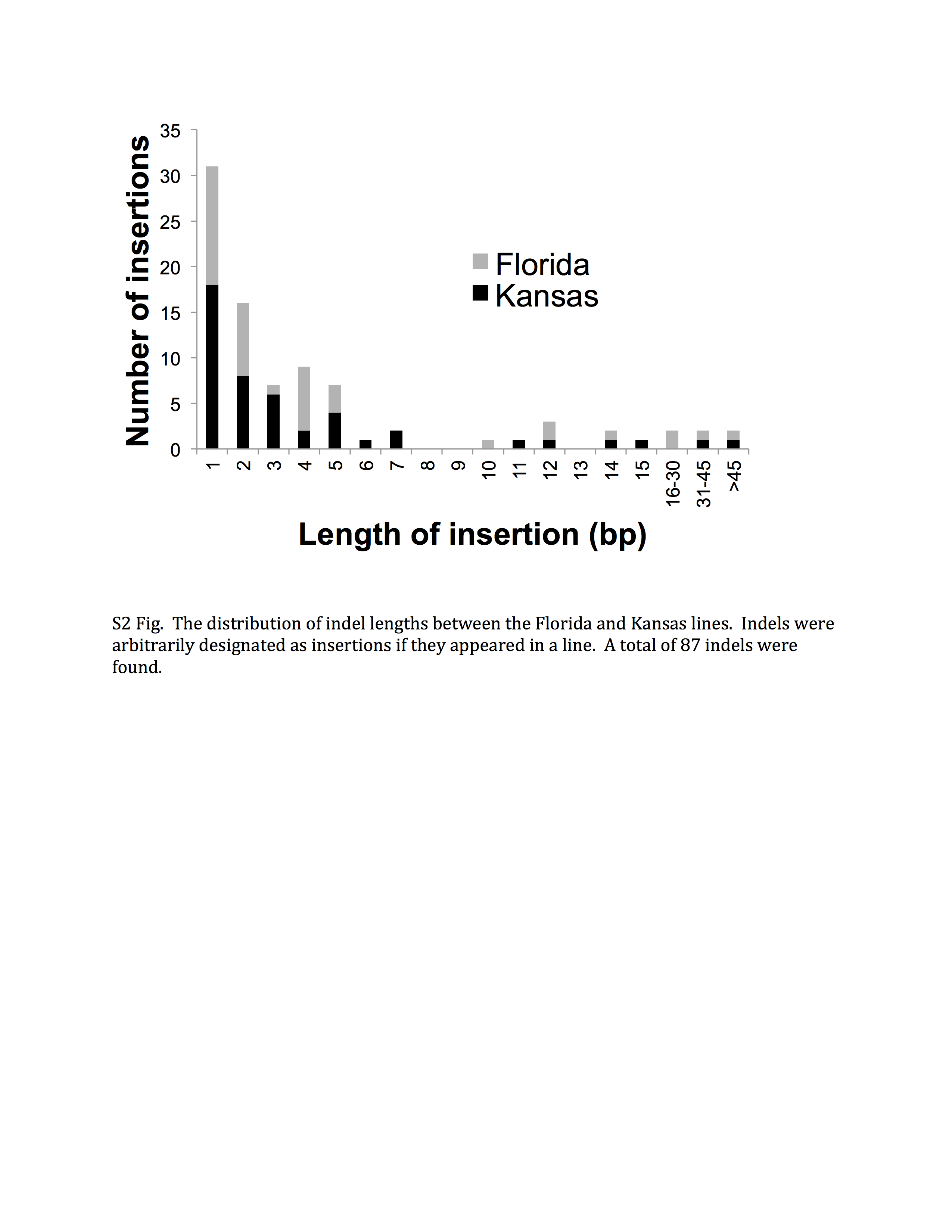

Supplement: S2 Fig — Indels were arbitrarily designated as insertions if they appeared in a line. A total of 87 indels were found. (TIFF) [file pone.0147014.s002.tiff]

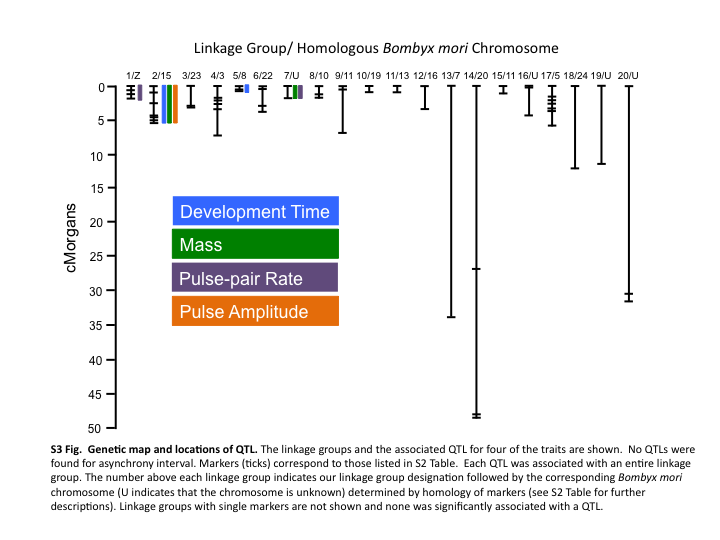

Supplement: S3 Fig — The linkage groups and the associated QTL for four of the traits are shown. No QTLs were found for asynchrony interval. Markers (ticks) correspond to those listed in Table B in S1 File. Each QTL was associated with an entire linkage group. The number above each linkage group indicates our linkage group designation followed by the corresponding Bombyx mori chromosome (U indicates that the chromosome is unknown) determined by homology of markers (see Table B in S1 File for further descriptions). Linkage groups with single markers are not shown and none was significantly associated with a QTL. (TIFF) [file pone.0147014.s003.tiff]
