# Supplementary material for: Development of a Genomic Resource and Quantitative Trait Loci Mapping of Male Calling Traits in the Lesser Wax Moth, Achroia grisella
Source: PLoS One. 2016 Jan 25;11(1):e0147014. doi: 10.1371/journal.pone.0147014 (PMC4726463; doi:10.1371/journal.pone.0147014)
Supplement: S1 File — (DOCX) [file pone.0147014.s004.docx]

S1 Table A. GO Slim Processes for the EST library

| Biological Process (GO level P) | N | % | Biological Process (GO level F) | N | % |
| --- | --- | --- | --- | --- | --- |
| Biological process | 27 | 10.59 | Molecular function | 35 | 16.51 |
| Translation | 23 | 9.02 | Ion binding | 22 | 10.38 |
| Transport | 15 | 5.88 | Oxidoreductase activity | 19 | 8.96 |
| Response to stress | 14 | 5.49 | Structural constituent of ribosome | 18 | 8.49 |
| Small molecule metabolic process | 14 | 5.49 | Transmembrane transporter activity | 12 | 5.66 |
| Signal transduction | 12 | 4.71 | Peptidase activity | 11 | 5.19 |
| Cellular protein modification process | 11 | 4.31 | RNA binding | 7 | 3.30 |
| Cellular nitrogen compound metabolic process | 10 | 3.92 | ATPase activity | 5 | 2.36 |
| Biosynthetic process | 9 | 3.53 | Kinase activity | 5 | 2.36 |
| Anatomical structure development | 6 | 2.35 | Lipid binding | 5 | 2.36 |
| Catabolic process | 6 | 2.35 | Structural molecular activity | 5 | 2.36 |
| Cell cycle | 6 | 2.35 | Enzyme regulator activity | 4 | 1.89 |
| Transmembrane transport | 6 | 2.35 | Ligase activity | 4 | 1.89 |
| Cytoskeleton organization | 5 | 1.96 | Nucleic acid binding | 4 | 1.89 |
| Generation of precursor metabolites and energy | 5 | 1.96 | Signal transducer activity | 4 | 1.89 |
| Cell differentiation | 4 | 1.57 | Translation factor activity, nucleic acid binding | 4 | 1.89 |
| Immune system process | 4 | 1.57 | DNA binding | 3 | 1.42 |
| Nucleobase-containing compound catabolic process | 4 | 1.57 | GTPase activity | 3 | 1.42 |
| Aging | 3 | 1.18 | Hydrolase activity | 3 | 1.42 |
| Carbohydrate metabolic process | 3 | 1.18 | Protein binding transcription factor activity | 3 | 1.42 |
| Cell death | 3 | 1.18 | 3-oxoacyl-[acyl-carrier-protein] reductase (NADPH) activity | 2 | 0.94 |
| Cell division | 3 | 1.18 | Isomerase activity | 2 | 0.94 |
| Cell-cell signaling | 3 | 1.18 | Metal ion binding | 2 | 0.94 |
| DNA metabolic process | 3 | 1.18 | Nucleotide binding | 2 | 0.94 |
| Embryo development | 3 | 1.18 | Nucleotidyltransferase activity | 2 | 0.94 |
| Homeostatic process | 3 | 1.18 | Unfolded protein binding | 2 | 0.94 |
| Metabolic process | 3 | 1.18 | Beta-N-acetylhexosaminidase activity | 1 | 0.47 |
| Oxidation-reduction process | 3 | 1.18 | Calcium ion binding | 1 | 0.47 |
| Protein folding | 3 | 1.18 | Catalytic activity | 1 | 0.47 |
| Autophagy | 2 | 0.78 | Copper ion binding | 1 | 0.47 |
| Chromosome organization | 2 | 0.78 | Cytoskeletal protein binding | 1 | 0.47 |
| Chromosome segregation | 2 | 0.78 | Electron carrier activity | 1 | 0.47 |
| Lipid metabolic process | 2 | 0.78 | Exonuclease activity | 1 | 0.47 |
| Mitotic nuclear division | 2 | 0.78 | Helicase activity | 1 | 0.47 |
| Neurological system process | 2 | 0.78 | Hydrolase activity, acting on glycosyl bonds | 1 | 0.47 |
| Phosphorylation | 2 | 0.78 | Hydrolase activity, hydrolyzing O-glycosyl compounds | 1 | 0.47 |
| Protein complex assembly | 2 | 0.78 | Lipid transporter activity | 1 | 0.47 |
| Proteoloysis | 2 | 0.78 | Lyase activity | 1 | 0.47 |
| Reproduction | 2 | 0.78 | Methyltransferase activity | 1 | 0.47 |
| Ribosome biogenesis | 2 | 0.78 | Microtubule binding | 1 | 0.47 |
| Cell adhesion | 1 | 0.39 | Omega peptidase activity | 1 | 0.47 |
| Cell motility | 1 | 0.39 | Phosphatase activity | 1 | 0.47 |
| Cell proliferation | 1 | 0.39 | Pyrroline-5-carboxylate reductase activity | 1 | 0.47 |
| Cellular amino acid biosynthetic process | 1 | 0.39 | Structural constituent of cytoskeleton | 1 | 0.47 |
| Cellular amino acid metabolic process | 1 | 0.39 | Transcription factor binding | 1 | 0.47 |
| Cellular component assembly | 1 | 0.39 | Transferase activity, transferring acyl groups | 1 | 0.47 |
| Cold acclimation | 1 | 0.39 | Transferase activity, transferring alkyl or aryl (other than methyl) groups | 1 | 0.47 |
| Cytoskeletal anchoring at plasma memberane | 1 | 0.39 | Transporter activity | 1 | 0.47 |
| Cytoskeleton-dependent intracellular transport | 1 | 0.39 | Ubiquitin thiolesterase activity | 1 | 0.47 |
| DNA replication | 1 | 0.39 | Zinc ion binding | 1 | 0.47 |
| Growth | 1 | 0.39 |  |  |  |
| L-proline biosynthetic process | 1 | 0.39 |  |  |  |
| Lipid transport | 1 | 0.39 |  |  |  |
| Microtubule cytoskeleton organization | 1 | 0.39 |  |  |  |
| Proline biosynthetic process | 1 | 0.39 |  |  |  |
| Ribonucleoprotein complex assembly | 1 | 0.39 |  |  |  |
| Ubiquitin-dependent protein catabolic process | 1 | 0.39 |  |  |  |
| Vesicle-mediated transport | 1 | 0.39 |  |  |  |
| Wnt signaling pathway | 1 | 0.39 |  |  |  |
| Total | 255 | 100.0 | Total | 212 | 100.0 |

S1 Table B. Correspondence of *Achroia grisella* markers to *Bombyx mori* scaffolds

| Locus name^a^ | Contig | Top BLASTX hit^b^ | Linkage group | *B. mori* scaffold^b^ | *B. mori* chromosome |
| --- | --- | --- | --- | --- | --- |
| C55 |  | guanine nucleotide-binding protein subunit beta 2-like | 1 | nscaf1690 | 1 |
| C151 |  | glutamine synthetase | 1 | nscaf1690 | 1 |
| C6n |  | NS | 1 | NS | 1 (inferred by linkage) |
| C19 | T4 | NS | 1 | NS | 1 (inferred by linkage) |
| C110 | T44 | ribosomal protein P0 | 2 | nscaf2655 | 15 |
| C214 |  | SCP-related protein | 2 | nscaf2655 | 15 |
| C121 |  | ribosomal protein P1 | 2 | nscaf2655 | 15 |
| 1E10 |  | extracellular sulfatase sulf-1 homolog | 2 | nscaf2887 | 15 |
| C172 |  | NS | 2 | NS | 15 (inferred by linkage) |
| 1G12 | T39 | Long wavelength-sensitive opsin | 2 | nscaf2888 | 15 |
| C88 |  | ribosomal protein partial | 2 | nscaf2888 | 15 |
| 2E03 |  | NS | 3 | NS | 23 (inferred by linkage) |
| C136 | T24 | achain crystal structure of juvenile hormone binding protein from *Galleria mellonella* hemolymph | 3 | nscaf3027 | 23 |
| 1E06 |  | Protein toll-like | 3 | nscaf3015 | 23 |
| C90 |  | Similar to CG7781 | 4 | nscaf2931 | 3 |
| C186 | T22 | control proteinHCTL024 | 4 | nscaf2930 | 3 |
| 4G05 |  | NS | 4 | NS | 3 (inferred by linkage) |
| C212 |  | Protein gar2 | 4 | nscaf2930 | 3 |
| C7 |  | NS | 4 | NS | 3 (inferred by linkage) |
| C124 |  | NS | 4 | NS | 3 (inferred by linkage) |
| C222 | T57 | Coil-coiled-helix-coil-coiled-helix domain-containing protein mitochondrial-like | 5 | nscaf2828 | 8 |
| 2H07 |  | Hypothetical protein KGM_18069 | 5 | nscaf2828 | 8 |
| C133 |  | Interferon-inducible double stranded RNA-dependent protein kinase activator A | 5 | nscaf2828 | 8 |
| 2H03 |  | NS | 6 | NS | 22 (inferred by linkage) |
| 4B05 |  | eukaryotic translation initiation factor 3 subunit g | 6 | nscaf1681 | 22 |
| 1A09 |  | arrestin-like protein | 6 | nscaf3005 | 22 |
| 3E12 |  | NS | 6 | NS | 22 (inferred by linkage) |
| C195 |  | NS | 7 | NS | Unknown |
| 2A03 | T17 | NS | 7 | NS | Unknown |
| C22 | T29 | Troponin C | 8 | nscaf2855 | 10 |
| C35 | T45 | Limbic system-assoicated membrane protein | 8 | nscaf2855 | 10 |
| 1E09 |  | Mical-like protein 2-like | 8 | nscaf2855 | 10 |
| 2G06 |  | DNA polymerase delta catalytic subunit | 8 | nscaf2860 | 10 |
| C43 |  | ubiquitin-conjugating enzyme e2 variant 2-like | 9 | nscaf3031 | 11 |
| C20 |  | NS | 9 | nscaf3031^d^ | 11 |
| C189 |  | Bc10 | 9 | NS | 11 (inferred by linkage) |
| 3C09 |  | Chemosensory protein | 10 | nscaf2767 | 19 |
| C156 | T28 | NS | 10 | NS | 19 (inferred by linkage) |
| C245 |  | Ribosomal protein l31 | 11 | nscaf1898 | 13 |
| C64 |  | lipopolysaccharide-induced tumor necrosis factor-alpha factor isoform x2 | 11 | nscaf1898 | 13 |
| C188 |  | Hypothetical protein KGM_15451 | 12 | nscaf556 | unknown |
| C145 | T33 | Sarcoplasmic calcium binding | 12 | nscaf3063 | 16 |
| C47 | T47 | RNA exonuclease 1-like protein | 13 | NS | 7 (inferred by linkage) |
| 1A12 |  | Probable gpi-anchored adhesin-like protein pga55 isoform x1 | 13 | nscaf2910 | 7 |
| 4G02 |  | NS | 14 | NS | 20 (inferred by linkage) |
| C161 |  | Carboxylesterase CarE-11 precursor | 14 | nscaf2780 | 20 |
| C119 |  | ubiquitin-conjugating enzyme e2 c | 14 | nscaf2937 | 20 |
| C140 | T41 | Tubulin beta-1 chain | 14 | nscaf2937 | 20 |
| 3F04 |  | aael008264-partial | 15 | nscaf3031 | 11 |
| 3H01 |  | NS | 15 | NS | 11 (inferred by linkage) |
| C139 |  | Ribosomal protein S19 | 16 | NS | Unknown |
| 2B01 |  | NS | 16 | NS | Unknown |
| C49 | T1 | NS | 16 | NS | Unknown |
| 4G04 |  | Small heat shock protein | 17 | nscaf2838 | 5 |
| C50 |  | arginine kinase | 17 | nscaf2838 | 5 |
| C98 |  | protein tyrosine phosphatase type iva 1 | 17 | nscaf2674 | 5 |
| C40 |  | serf-like protein | 17 | NS | 5 (inferred by linkage) |
| 4C10 |  | Pre-mRNA branch site p14-like | 17 | NS | 5 (inferred by linkage) |
| C160 |  | 14-3-3-zeta | 17 | nscaf2529 | 5 |
| C2 | T43 | H+ transporting ATP subunit g | 17 | nscaf3075 | 5 |
| C39 | T48 | thioredoxin-like protein | 18 | nscaf2891 | 24 |
| 3H02 |  | short-chain dehydrogenase | 18 | nscaf2891 | 24 |
| C30 | T32 | Microtubule associated protein futsch | 19 | NS | Unknown |
| C228 | T34 | NS | 19 | NS | Unknown |
| 1D03 |  | NS | 20 | NS | Unknown |
| 2G09 |  | NS | 20 | NS | Unknown |
| C16 | T2 | Unknown | 20 | NS | Unknown |
| 1A06 |  | Tail protein | 21 | nscaf2837 | 27 |
| C126 |  | prefoldin subunit 5-like | 22 | nscaf2853 | 6 |
| C93 |  | NS | 23 | NS | Unknown |
| C66 | T20 | ADP/ATP translocase | 24 | nscaf2686 | 24 |
| 1D10 |  | heat shock protein | 25 | nscaf1898 | 13 |
| C4 |  | peroxiredoxin 5 | 26 | nscaf3150 | Unknown |
| C157 |  | NS | 27 | NS | Unknown |
| 2F11 |  | NS | 28 | NS | Unknown |

^a^Within each linkage group, markers are listed in map order

^b^NS indicates that no significant match was found.

^c”^Unknown” indicates that either a scaffold has not been placed, or that a locus did not have a significant Blastx hit.

^d^In this one case, there was not a significant Blastx hit using our cutoff criteria, but in a Blastx search to the Silkworm Database (http://www.silkdb.org/) a significant scaffold was found.
